# Fracture Patterns and Peripheral Brachial Plexus Injury in Humerus Fractures: A Retrospective Study

**DOI:** 10.1055/a-2596-1347

**Published:** 2025-08-19

**Authors:** Mohammed Musaed Almalki, Bander S. Alrashedan, Ahmed Shaya Alragea, Adel Faiz Alshihri, Hani S Serhan, Husam Fahmi AlFashtaki

**Affiliations:** 1 Department of Orthopedic Surgery, King Saud Medical City, Riyadh, Kingdom of Saudi Arabia; 2 Department of Orthopedic Surgery, King Salman Armed Forces Hospital, Tabuk, Kingdom of Saudi Arabia

**Keywords:** humeral shaft fracture, radial nerve palsy, fracture pattern, retrospective cohort, trauma severity, biomechanics

## Abstract

**Background:**

Peripheral brachial plexus injuries, particularly Radial nerve palsy (RNP), are a common complication of humeral shaft fractures. Despite previous research, the specific fracture patterns associated with RNP remain unclear.

**Objectives:**

This study aims to assess the frequency and patterns of humeral shaft fractures, determine the incidence of peripheral brachial plexus injuries such as radial and ulnar nerve palsies, and investigate the association between specific fracture patterns and these nerve injuries. We also explored other factors influencing RNP development and identified potential risk factors or predictors.

**Methods:**

This retrospective cohort study analyzed 144 patients with humeral shaft fractures at King Saud Medical City from 2015 to 2022. Patient data, such as age, gender, and neurovascular status, were extracted and analyzed using SPSS. RNP was diagnosed clinically. Statistical analyses included chi-square and student *t* -tests, with a *p* -value of <0.05 for significance.

**Results:**

This study found a 37.5% incidence of RNP in humeral shaft fractures. Significant differences were observed in age ( *p* = 0.032) and mechanism of injury ( *p* < 0.0001), with most fractures due to road traffic accidents (72.22%) and closed (93.75%). Common fracture patterns were AO 12A (37.5%) and AO 12B (39.58%), with a higher prevalence of comminuted fractures (71 cases). Significant differences in fracture patterns ( *p* < 0.0001) and anteromedial comminution ( *p* = 0.002) were noted between patients with and without RNP. Interestingly, four cases of concomitant ulnar nerve palsy were observed in patients with radial nerve palsy. However, no significant differences were found in gender ( *p* = 0.343), open fractures ( *p* = 0.214), or associated fractures ( *p* = 0.106).

**Conclusion:**

This study suggests that the severity of trauma, rather than specific fracture patterns, is a more significant factor in RNP development in humeral shaft fractures. Further research is needed to understand the underlying biomechanics.

## Introduction

The radial nerve is one of the terminal branches that originated from the posterior cord of the brachial plexus. Its unique course in the arm predisposes it to a higher chance of injury. In the arm, it traverses the humerus from medial to lateral, around 20 cm from the medial epicondyle and 14 cm from the lateral epicondyle. After traversing the humerus, the nerve will enter the lateral intermuscular septum and travel posterolateral to the humerus and elbow joint. 1 2

According to some studies, radial nerve palsy (RNP) estimates are around 8.5 to 11.8% in all humeral shaft fractures. 3 4 5 6 7 8 9 As expected, most of the RNP results from middle and distal shaft fracture of the humerus. It is hypothesized that spiral distal humerus fracture, known as Holstein-Lewis fracture, has the highest rate of RNP since the nerve lies in direct contact with the lateral cortex of the humerus. This is due to distal fragment lateral displacement, which can lead the radial nerve to be lacerated or entrapped between the bone fragments. 10 In a study done by Shao et al, 5 the authors suggested that the connection between Holstein–Lewis fractures and RNP may not be as significant as initially thought. Their systematic review indicates that the area of risk encompasses both the middle and distal parts of the shaft. This was also implied in a study by Ekholm et al, 11 where he found that Holstein–Lewis fracture and other simple fracture patterns, such as transverse and oblique, are equal in causing RNP.

Biomechanically, the fracture happens because of significant compression on one side of the cortex, which in turn will lead to tension in the other cortex. In pediatric supracondylar humerus fracture, the presence of RNP is associated with fracture pattern. Posteromedial displacement results in higher RNP. 12 13 This is attributed to the biomechanics of the fracture, whereas posteromedial displacement will put the lateral structures in tension. This is also seen with distal femur physeal fracture, where the tension side usually has entrapped soft tissue (periosteum) and the metaphyseal comminution on the compression side. 14

Despite these insights, there remains a knowledge gap in our understanding of the specific fracture patterns in humeral shaft fractures predisposing to RNP. This study aims to examine the association between various fracture patterns in the humeral shaft and the occurrence of RNP, thus providing a clearer understanding of the mechanisms behind this complication.

## Methods

### Study Design

This is a retrospective cohort study conducted at King Saud Medical City (KSMC), which is a level I Trauma center located in the central region of Riyadh, Saudi Arabia. It is considered the largest Ministry of Health Community trauma center and destination for referral to secondary hospitals. We included all cases presented to our hospital from January 1, 2015, to June 2022, with the diagnosis of humerus shaft fractures after obtaining approval from the Institutional Review Board (IRB) of KSMC. The primary aim of the study is to assess the pattern of fracture and its association with RNP. The secondary aim of the study was to assess the association between the mechanism of injury and RNP and the association of other nerve palsy, such as ulnar and median nerves.

### Patient Selection

The included sample consisted of cases that presented acutely with humeral diaphyseal fracture (AO/OTA 12 A, B, and C) above the age of 14 years old. Cases excluded were pathological fractures, metaphyseal fractures, or patients who were lost to follow-up. We found 144 cases that met our criteria.

### Data Collection

**Fig. 1:**
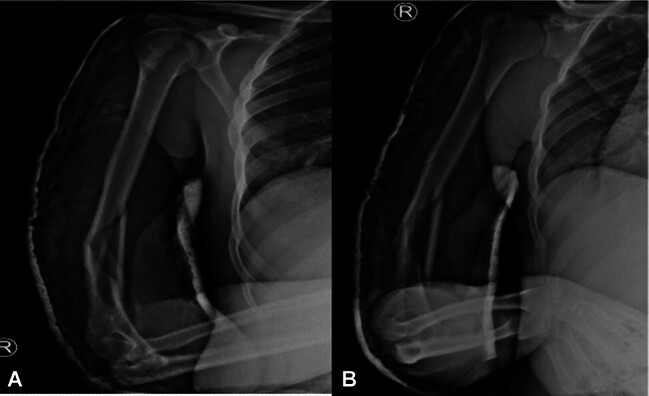
( **A** ) AP view of humerus showing anteromedial comminution. ( **B** ) Lateral view of the humerus with anteromedial comminution.

The patient's files were accessed through the medical records department after approval from the IRB (reference number: H1RI-31-Aug23–02). Data such as age, gender, neurovascular status, specifically RNP, mechanism of injury, and presence of open or closed fracture were extracted. Picture archiving and communication system (PACS) was used to analyze the imaging of humerus shaft fractures. Humerus shaft fractures were classified based on AO/OTA classification. Fractures were stratified based on the location of the comminution into anteromedial, anterolateral, posteromedial, posterolateral, and no comminution ( Figs. 1 2 3 4 ). RNP was diagnosed clinically by neurological examinations. RNP was defined as wrist and/or finger drop and or sensory deficit at the radial nerve territory. In addition, ulnar nerve palsy was identified as an inability to abduct the fingers and sensory deficit at the ulnar nerve territory.

**Fig. 2:**
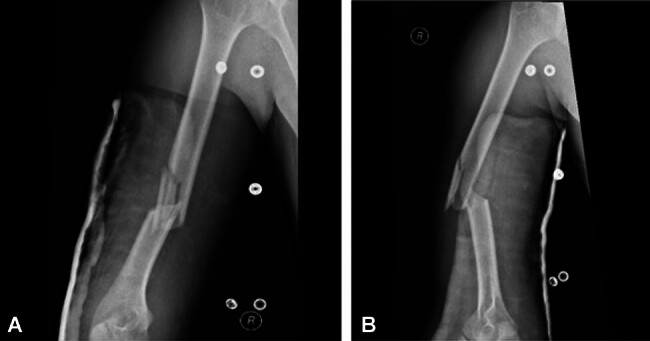
( **A** ) AP view of humerus showing anterolateral comminution. ( **B** ) Lateral view showing anterolateral comminution.

**Fig. 3:**
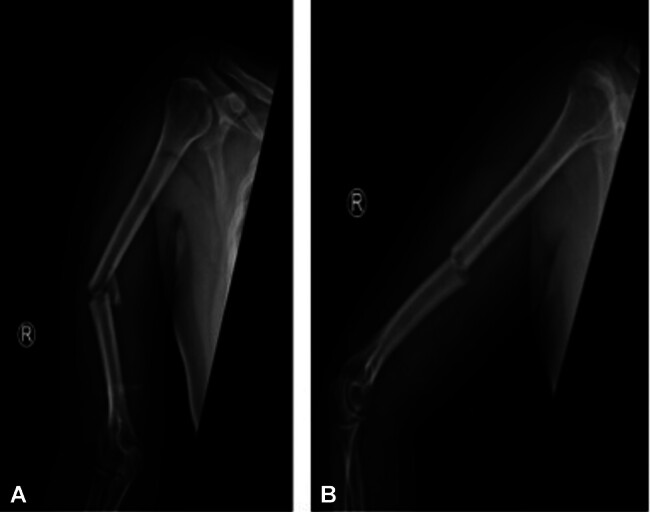
( **A** ) Shows AP view with posteromedial comminution. ( **B** ) Lateral view showing posteromedial comminution.

**Fig. 4:**
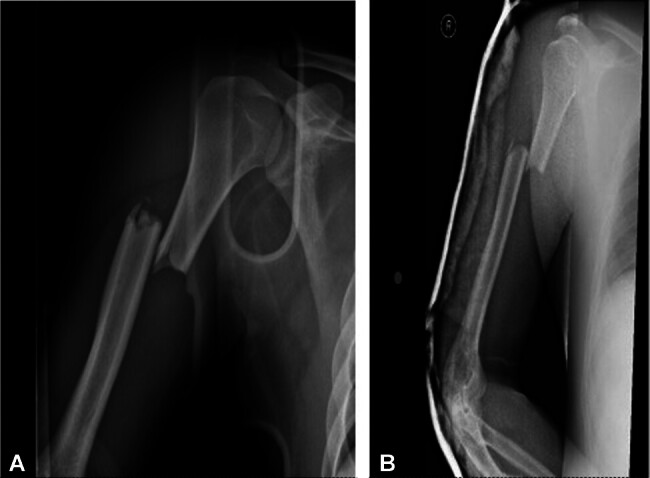
( **A** ) AP view showing posterolateral humerus comminution. ( **B** ) Lateral view showing posterolateral humerus comminution.

### Statistical Analysis

All cases were managed and followed at our hospital. Data were collected in an Excel sheet and analyzed using SPSS software (V.21.0, IBM Corp.). Patients were stratified into two groups, whether patients sustained RNP or not. Analysis of demographic data, including age, gender, and data such as the presence of open fracture, mechanism of injury, fracture pattern, and location of comminution, was done between the groups to see any differences that could affect the results when compared with the primary outcome. Categorical values were analyzed using the chi-square test. Numerical values were analyzed using a student *t* -test when two groups were compared. Data normality was assessed using the Shapiro–Wilk test. A *p* -value of less than 0.05 is considered statistically significant.

## Results

The study included 144 cases: 18 (12.5%) females and 126 (87.5%) males. Age ranged from 16 to 78 years, with a mean age of 32.7 years. Out of 144 cases, there were 54 (37.5%) patients who sustained RNP, 4 of which had concomitant ulnar nerve palsy, and 90 (62.5%) patients who did not have RNP. Around 104 (72.22%) cases sustained a fracture due to RTA, 14 (9.72%) cases due to crush injury, and 26 (18.06%) cases due to fall. Of all fractures, 135 (93.75%) cases were closed fractures, and 9 (6.25%) cases were open. There were 54 (37.5%) cases of type AO 12A, 57 (39.58%) cases were AO 12B, and 33 (22.92%) cases were AO12C. Seventy-one (49.3%) cases were comminuted, and 73 (50.7%) cases were simple fracture cases ( Table 1 ).

**Table 1:** Baseline characteristics for patients included in the study

Variable	Number of cases
Total cases	144
Females (%)	18 (12.5%)
Males (%)	126 (87.5%)
Radial nerve palsy (%)	54 (37.5%)
No radial nerve palsy (%)	90 (62.5%)
Mechanism of injury	
RTA (%)	104 (72.2%)
Crush (%)	14 (9.72%)
Fall (%)	26 (18.06%)
Closed fracture (%)	135 (93.75%)
Open fracture (%)	9 (6.25%)
AO/OTA classification	
AO 12A (%)	51 (35.41%)
AO 12B (%)	57 (36.11%)
AO 12C (%)	20 (13.88%)
AO 13A (%)	5 (3.47%)
AO 13B (%)	4 (2.77%)
AO 13C (%)	12 (8.33%)
Fracture pattern	
Comminuted (%)	71 (49.3%)
Noncomminuted (%)	73 (50.7%)

When testing for RNP and the association between the variables. The group without palsy included 80 males (88.89%) and 10 females (11.11%), while the RNP group had 46 males (85.19%) and 8 females (14.81%), showing no significant gender difference ( *p* = 0.343). The average age was 34.5 years (SD: 15.26) for the nonpalsy group and 29.89 years (SD: 10.17) for the palsy group ( *p* = 0.032). In the nonpalsy group, there were 74 RTA cases (82.22%), no crush injuries (0%), and 17 falls (18.89%). The palsy group had 30 RTA cases (55.56%), 14 crush injuries (25.93%), and 9 falls (16.67%; *p* < 0.0001). Open fractures were less common, with four cases (4.44%) in the nonpalsy group and five cases (9.26%) in the palsy group ( *p* = 0.214). The fracture pattern analysis for patients showed a statistically significant difference ( *p* < 0.0001). Comminution was observed in 40 patients (44.44%) without palsy and 31 (57.4%) with palsy ( *p* = 0.169; Table 2 ).

**Table 2:** Association of demographic, clinical, and radiographic factors with associated nerve palsy in patients with humerus fracture

Associated nerve palsy	Yes ( *n* = 54)	No ( *n* = 90)	*p* -Value
Male:Female (%)	46 (85.19%):8 (14.81%)	80 (88.89%):10 (11.11%)	0.343
Age	29.89 (SD: 10.17)	34.5 (SD: 15.26)	*p* = 0.032
Mechanism of injury (%)	RTA: 30 (55.56%) Crush: 14 (25.93%) Fall: 9 (16.67%)	RTA: 74 (82.22%) Crush: 0 (0%) Fall: 17 (18.89%)	<0.0001
Open vs. closed fracture (%)	5 open (9.26%) 48 closed (90.74%)	4 open (4.44%) 87 closed (95.56%)	0.214
Fracture pattern (%)	AO 12A: 15 cases (27.77%) AO 12B: 16 cases (29.62%) AO 12C: 16 cases (29.62%) AO 13A: 3 cases (5.55%) AO 13B: 2 cases (3.70%) AO 13C: 2 cases (3.70%)	AO 12A: 36 cases (40%) AO 12B: 36 cases (40%) AO 12C: 4 cases (4.44%) AO 13A: 2 cases (2.22%) AO 13B: 2 cases (2.22%) AO 13C: 10 cases (11.11%)	<0.0001
Presence of comminution (%)	Comminution 31 (57.4%) No comminution 23 (42.6%)	Comminution 40 (44.44%) No comminution 50 (55.56%)	0.169

When analyzing we found that fracture comminution pattern was associated with RNP when we compared RNP cases to the cases who did not sustain nerve palsy ( *p* = 0.002). For instance, medial comminution was found to be significantly higher in RNP group (46.29%) compared to non-radial nerve palsy group (28.8%). In more details, patients without radial nerve palsy ( *n* = 90), 47 had no comminution (52.22%), 11 had posteromedial (12.22%), 15 anteromedial (16.67%), 2 anterolateral (2.22%), and 15 posterolateral (16.67%) comminutions. In the palsy group ( *n* = 54), 22 had no comminution (40.74%), 4 posteromedial (7.41%), 21 anteromedial (38.89%), 5 anterolateral (9.26%), and 2 posterolateral (3.70%) ( *p* = 0.002). Regarding fractures, 17 without palsy (18.89%) and 17 with palsy (31.48%) had associated fractures, whereas 73 without (81.11%) and 37 with palsy (68.52%) did not ( *p* = 0.106).

**Table 3:** Fracture characteristics

Associated nerve palsy	Yes ( *n* = 54)	No ( *n* = 90)	*p* -Value
Comminution	No = 22 Posteromedial = 4 Anteromedial = 21 Anterolateral = 5 Posterolateral = 2	No = 47 Posteromedial = 11 Anteromedial = 15 Anterolateral = 2 Posterolateral = 15	0.002
Associated fractures. No associated fractures	17 37	17 73	0.106

## Discussion

In the study, significant findings included a higher incidence of RNP in younger patients, with an average age of 29.89 years in the palsy group compared with 34.5 years in the nonpalsy group. The cause of fractures varied significantly between groups, with road traffic accidents more prevalent in the nonpalsy group and crush injuries more common in the palsy group. Fracture patterns showed a notable difference; a more even distribution across AO types was observed in the palsy group, suggesting a strong association between AO 12C and the occurrence of RNP. Additionally, there was a significant variation in the location of comminution, particularly the higher incidence of anteromedial comminution in the palsy group. However, associated fractures did not show a significant difference between the groups.

Biomechanically, the butterfly fragment happens due to both compression and bending forces. If the bending forces start laterally, then the compression and comminution will be on the medial aspect of the humerus. 15 As a result, the fracture spike will happen on the lateral aspect of the humerus, which predisposes the radial nerve to injury. Our study found that medially sided comminution was associated with RNP in 46% (25 cases) and 28% (26 cases) of the nonpalsy group. This suggests that the location of the comminution is in fact related to RNP. Shao et al and Ekholm et al 5 10 found that the most common fracture patterns associated with RNP were simple fracture patterns such as transverse, oblique, and spiral fractures. Similarly, Khan et al conducted a retrospective study encompassing 80 patients with closed humeral shaft fractures; they found out the prevalence of RNP was around 8.75% (7/80), with most fractures spiral. 16 In our study, we did find differences regarding fracture comminution between RNP and non-palsy group and these findings suggest that medial-sided comminution could be risk factor for RNP.

In our study, we observed a strong correlation between RNP and high-energy injury mechanisms. Specifically, all cases of crush injuries (14 in total) in our study were linked with RNP. This observation aligns with findings from Kong et al, 9 who reported a 26.6% incidence of RNP associated with high-energy injuries. Furthermore, while the incidence of RNP in existing literature typically ranges from 8.5 to 11.8%, our study recorded a significantly higher prevalence of 37.5% (54/144 cases). This notable increase can likely be attributed to our study in a trauma center, where most cases involved high-energy injuries. Additionally, several studies regarding RNP found that the prevalence is significantly higher in population-sustained humeral shaft fractures due to high energy. For instance, Entezari et al found that 68% of all high-energy humeral shaft fracture cases sustained RNP. 17 Similarly, Lang et al reported that 52% of cases sustained RNP, 18 and Claessen et al reported 66 RNP cases out of 325 high-energy humeral shaft fractures. 19 This led both to conclude that the high energy mechanism of injury is the cause of RNP. As a result, we believe that certain fracture patterns are more prevalent in high-energy traumas rather than being directly linked to RNP. Hence, it is the intensity of the injury that increases the risk of RNP, rather than a specific pattern of fracture being a direct cause of RNP.

Interestingly, we found four cases that had concomitant ulnar nerve palsy with RNP. These cases were a result of high-energy trauma and were not associated with specific fracture patterns. Specifically, three occurred following RTA, while one was due to a crush injury. Two of the fractures were comminuted, and the remaining had simple fracture patterns. Pathak et al and Stahl et al 20 21 found similar cases of ulnar nerve palsy in humeral shaft fracture, and both cases were attributed to the mechanism of injury as the culprit in the nerve palsy.

While this study offers valuable insights into the relationship between humerus fractures and RNP, it is essential to acknowledge inherent methodological limitations. Additionally, the study's single-center nature and the sample size may restrict the generalizability of the findings to broader populations. Despite these limitations, our study adds to what we know about RNP in humeral fractures and suggests areas for more research.

## Conclusion

In conclusion, this study sheds significant light on the complex relationship between RNP and humeral shaft fractures. Our findings suggest a complex interplay between the type of fracture, the mechanism of injury, and the occurrence of RNP. Our investigation reveals a higher RNP prevalence in high-energy trauma cases, such as crush injuries, challenging the widely held belief that specific fracture patterns, such as Holstein–Lewis fractures, predominantly cause RNP. The data points to the severity and nature of the trauma as more influential factors in RNP likelihood than the fracture pattern itself. These insights emphasize the importance of a deeper understanding of humeral shaft fracture biomechanics and their impact on nerve injury. The study's findings pave the way for further research in this domain, potentially leading to improved treatment strategies and better patient outcomes in humeral fractures.

## References

[1] Bumbasirevic M Palibrk T Lesic A Atkinson H Radial nerve palsy EFORT Open Rev 2017 1 08 286 294 28461960 10.1302/2058-5241.1.000028 PMC5367587

[2] Latef T J Bilal M Vetter M Iwanaga J Oskouian R J Tubbs R S Injury of the radial nerve in the arm: a review Cureus 2018 10 02 e2199 29666777 10.7759/cureus.2199 PMC5902095

[3] Ekholm R Adami J Tidermark J Hansson K Törnkvist H Ponzer S Fractures of the shaft of the humerus. An epidemiological study of 401 fractures J Bone Joint Surg Br 2006 88 11 1469 1473 17075092 10.1302/0301-620X.88B11.17634

[4] Noble J Munro C A Prasad V SSV Midha R Analysis of upper and lower extremity peripheral nerve injuries in a population of patients with multiple injuries J Trauma 1998 45 01 116 122 9680023 10.1097/00005373-199807000-00025

[5] Shao Y C Harwood P Grotz M RW Limb D Giannoudis P V Radial nerve palsy associated with fractures of the shaft of the humerus: a systematic review J Bone Joint Surg Br 2005 87 12 1647 1652 16326879 10.1302/0301-620X.87B12.16132

[6] Belayneh R Lott A Haglin J Konda S Leucht P Egol K Final outcomes of radial nerve palsy associated with humeral shaft fracture and nonunion J Orthop Traumatol 2019 20 01 18 30923949 10.1186/s10195-019-0526-2 PMC6439110

[7] Ring D Chin K Jupiter J B Radial nerve palsy associated with high-energy humeral shaft fractures J Hand Surg Am 2004 29 01 144 147 14751118 10.1016/j.jhsa.2003.09.013

[8] Han S-H Cho J-W Ryu H-S Treatment of radial nerve palsy associated with humeral shaft fracture Archives Hand Microsurg 2020 25 01 60 66

[9] Kong C G Sur Y J Jung J W Park H Y Primary radial nerve palsy associated with humeral shaft fractures according to injury mechanism: is early exploration needed? J Shoulder Elbow Surg 2021 30 12 2862 2868 34411723 10.1016/j.jse.2021.07.013

[10] Ekholm R Ponzer S Törnkvist H Adami J Tidermark J The Holstein-Lewis humeral shaft fracture: aspects of radial nerve injury, primary treatment, and outcome J Orthop Trauma 2008 22 10 693 697 18978544 10.1097/BOT.0b013e31818915bf

[11] Ekholm R Ponzer S Törnkvist H Adami J Tidermark J Primary radial nerve palsy in patients with acute humeral shaft fractures J Orthop Trauma 2008 22 06 408 414 18594306 10.1097/BOT.0b013e318177eb06

[12] Valencia M Moraleda L Díez-Sebastián J Long-term functional results of neurological complications of pediatric humeral supracondylar fractures J Pediatr Orthop 2015 35 06 606 610 25379825 10.1097/BPO.0000000000000337

[13] Babal J C Mehlman C T Klein G Nerve injuries associated with pediatric supracondylar humeral fractures: a meta-analysis J Pediatr Orthop 2010 30 03 253 263 20357592 10.1097/BPO.0b013e3181d213a6

[14] Rockwood and Wilkins' Fractures in Children - Google Books . Accessed October 15, 2024 at:https://books.google.com.pk/books?hl=en&lr=&id=QVIdXV_F8M4C&oi=fnd&pg=PA24&dq=Price+CT,+Herrera-Soto+J:+Extra-articular+injuries+of+the+knee,+in+Beaty+JH,+Kasser+JR,+eds:+Rockwood+and+Wilkins%E2%80%99+Fractures+in+Children,&ots=Bi3osgFN0B&sig=AsAxx-uAKaHv733qT80WBNjdvFI&redir_esc=y#v=onepage&q&f=false

[15] Rockwood and Green's Fractures in Adults - Google Books. Accessed October 15, 2024 at:https://www.google.com.pk/books/edition/Rockwood_and_Green_s_Fractures_in_Adults/UOpkN2i5Y6sC?hl=en&gbpv=1&dq=Rockwood+%26+Green%E2%80%99s+Fractures+in+Adults.+9th+edition&printsec=frontcover

[16] Khan M M Ali M Girma J Faheem M U Jadoon A Aziz A Radial nerve injury in patients with closed fracture of humerus shaft in high energy trauma cases J Ayub Med Coll Abbottabad 2022 34 04 S1000 S1002 10.55519/JAMC-04-S4-11082 36550662

[17] Entezari V Olson J J Vallier H A Predictors of traumatic nerve injury and nerve recovery following humeral shaft fracture J Shoulder Elbow Surg 2021 30 12 2711 2719 33964428 10.1016/j.jse.2021.04.025

[18] Lang N W Ostermann R C Arthold C Joestl J Platzer P Retrospective case series with one year follow-up after radial nerve palsy associated with humeral fractures Int Orthop 2017 41 01 191 196 27079837 10.1007/s00264-016-3186-3

[19] Claessen F MAP Peters R M Verbeek D O Helfet D L Ring D Factors associated with radial nerve palsy after operative treatment of diaphyseal humeral shaft fractures J Shoulder Elbow Surg 2015 24 11 e307 e311 26341025 10.1016/j.jse.2015.07.012

[20] Pathak R Kalakoti P Prasad D V Peeyuusha D Sharma R Ulnar nerve injury after a comminuted fracture of the humeral shaft from a high-velocity accident: a case report J Med Case Rep 2012 6 192 22781595 10.1186/1752-1947-6-192 PMC3419694

[21] Stahl S Rosen N Moscona R Ulnar nerve palsy following fracture of the shaft of the humerus J Orthop Trauma 1998 12 05 363 364 9671191 10.1097/00005131-199806000-00013

